# *OsRCI-1*-Mediated GLVs Enhance Rice Resistance to Brown Planthoppers

**DOI:** 10.3390/plants13111494

**Published:** 2024-05-29

**Authors:** Kaiming Mao, Chengzhe Li, Huacai Zhai, Yuying Wang, Yonggen Lou, Wenhua Xue, Guoxin Zhou

**Affiliations:** 1 Key Lab for Biology of Crop Pathogens and Insect Pests and Their Ecological Regulation of Zhejiang Province, The Key Laboratory for Quality Improvement of Agricultural Products of Zhejiang Province, College of Advanced Agricultural Sciences, Zhejiang A & F University, Hangzhou 311300, China; mkm759288843@163.com (K.M.); chengzheli@163.com (C.L.); huacaizhai@163.com (H.Z.); wyy3112666500@163.com (Y.W.); 2 State Key Laboratory of Rice Biology, Ministry of Agriculture Key Lab of Molecular Biology of Crop Pathogens and Insects, Institute of Insect Sciences, Zhejiang University, Hangzhou 310058, China; yglou@zju.edu.cn

**Keywords:** rice, green leaf volatiles (GLVs), *cis*-3-hexen-1-ol, *cis*-3-hexenal, *Nilaparvata lugens*, *Anagrus nilaparvatae*

## Abstract

Green leaf volatiles (GLVs) play pivotal roles in plant anti-herbivore defense. This study investigated whether the rice 13-lipoxygense gene *OsRCI-1* is involved in GLV production and plant defense in rice. The overexpression of *OsRCI-1* (*oeRCI* lines) in rice resulted in increased wound-induced levels of two prominent GLVs, *cis*-3-hexen-1-ol and *cis*-3-hexenal. In a previous study, we found that the overexpression of *OsRCI-1* reduced the colonization by the rice brown planthopper (BPH, *Nilaparvata lugens*) but increased the attractiveness to the egg parasitoid *Anagrus nilaparvatae* compared to wild-type (WT) plants. This study found that when *cis*-3-hexen-1-ol, but not *cis*-3-hexenal, was added to WT plants, it could change the BPH’s colonization preference, i.e., more BPHs preferred to colonize the *oeRCI* lines. The exogenous application of *cis*-3-hexen-1-ol or *cis*-3-hexenal to BPH-infested WT plants could weaken or overturn the preference of *A. nilaparvatae* for *oeRCI* lines. However, field experiments revealed that only *cis*-3-hexenal was attractive to the parasitoid and increased the parasitism rates of BPH eggs. These results indicate that *OsRCI-1* is involved in rice GLV production and therefore modulates both direct and indirect defense in rice.

## 1. Introduction

Lipoxygenase (LOX), the pivotal enzyme within the fatty acid metabolism pathway, can be divided into *9-LOXs* and *13-LOXs* based on the position of oxygenation in the substrate carbon chain, participating in various aspects of plant growth, development, and responses to both biotic and abiotic stresses [1]. Two branch pathways of the LOX pathway, the allene oxide synthase pathway (AOS) and the hydroperoxide lyase pathway (HPL), have been well studied in induced plant defense against herbivores. Green leaf volatiles (GLVs) including C6 alcohols, aldehydes, and esters, acting through the HPL pathway, play important roles in plant resistance against herbivores, as well as the well-known signal molecules, jasmonates (JAs), produced from the AOS pathway [2].

GLVs, known for their distinct and strong aromas, reminiscent of fresh grass and green leaves, are mostly produced during the early stage of plant development and are rapidly released when cells are mechanically damaged or exposed to herbivores for feeding or oviposition [3]. Research shows that GLVs play a crucial role in both direct and indirect plant defense against insect pests [4]. In particular, emissions of the GLV compound *cis*-3-hexen-1-ol from plants such as rice (*Oryza sativa* L.; Gramineae: Oryza), tomato (*Solanum lycopersicum* L.; Solanaceae: *Solanum*), and tea (*Camellia sinensis* (L.) Kuntze; Theaceae: Camellia) have been shown to decrease pest numbers by attracting their natural enemies [5,6,7]. Specifically, studies focusing on key genes of the oxylipin pathway, such as rice *OsPLDa4*, *-a5*, and *OsHPL3*, have shown that *cis*-3-hexen-1-ol, released by rice plants, acts as a repellent against the rice brown planthopper (BPH, *Nilaparvata lugens* Stål; Hemiptera: Delphacidae), while attracting its natural enemy [5,8]. In contrast, the release of *cis*-3-hexenal from rice plants was found to reduce their resistance to the white-backed planthopper (WBPH, *Sogatella furcifera* Horváth; Hemiptera: Delphacidae) [9].

In rice, 14 LOX genes have been identified, of which seven genes have been cloned and characterized, including *OsRLL/OsHI-LOX*, *LOX-2*, *LOX-3*, *Osr9-LOX1*, *OsRCI-1*, and *OsLOX1* [10,11,12,13,14,15]. The resistance of rice against the rice brown planthopper was reduced in the antisense-suppressed lines of *OsLOX1*, while it was enhanced in the overexpression lines, and *OsLOX1* was related to the synthesis of JA and *cis*-3-hexen-1-ol [16]. Zhou et al. (2009) cloned the *OsHI-LOX* gene from the rice *13-LOXs*, and the antisense-suppressed mutant rice exhibited enhanced resistance to piercing-sucking brown planthoppers but reduced resistance to chewing insects like the striped stem borer (*Chilo suppressalis* Walker; Lepidoptera: Pyralidae) and rice leaf folder (*Cnaphalocrocis medinalis* Guenée; Lepidoptera: Pyralidae), but they found that *OsHI-LOX* was only involved in the synthesis of JA, rather than the production of GLVs [13]. *OsRCI-1* has been identified as a member of the *13-LOX* family [15]. *OsRCI-1* is induced by compounds such as benzo-(1,2,3)-thiadiazole-7-carbothioic S-methyl ester (BTH) and benzo-(1,2,3)-thiadiazole-7-carbothioic S-methyl ester (INA). The overexpression of *OsRCI-1* in rice transiently resulted in the upregulation of the pathogenesis-related protein PR1 gene [17]. *OsRCI*-*1* is also reported to be involved in BPH-induced JA bursts and plays a positive role in rice defense against BPH [18].

The biosynthesis of GLVs is orchestrated through the oxygenation of acyl groups by pathway-specific lipoxygenases; subsequent cleavage reactions facilitated by hydroperoxide lyases contribute to the formation of these characteristic volatile compounds [19]. In this study, we used overexpression *OsRCI-1* rice (*oeRCI* lines) [18] to investigate whether *OsRCI-1* was involved in rice GLV production and whether these GLVs played a role in the regulation of BPH colonization and the behavior of its egg parasitoid wasp, *Anagrus nilaparvatae* Pang et Wang (Hymenoptera: Mymaridae). We hypothesized that the overexpression of *OsRCI-1* in rice would enhance the production of GLVs, therefore playing a role in regulating BPH colonization and the behavior of its egg parasitoid *A. nilaparvatae*.

## 2. Results

### 2.1. Overexpressing OsRCI-1 Enhances Production of Mechanical Damage-Induced GLVs in Rice

**Figure 1:**
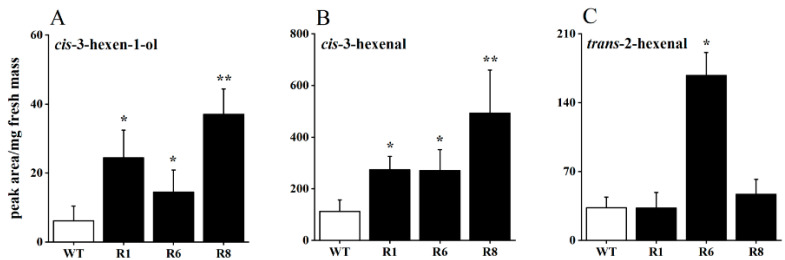
The content of *cis*-3-hexen-1-ol (**A**), *cis*-3-hexenal (**B**), and *trans*-2-hexenal (**C**) in wild-type (WT) and *oeRCI* lines (R1, R6, and R8) after mechanical damage treatment. Asterisks indicate significant differences between pairs of samples within time points (*, *p* < 0.05; **, *p* < 0.01; Student’s *t*-test).

To investigate the impact of *OsRCI-1* on the production of rice GLVs, the levels of GLVs from *OsRCI-1* overexpression rice (*oeRCI* lines) and wild-type rice plants were estimated after mechanical damage treatment. After the mechanical damage treatment, the content of three GLVs showed varying degrees of increase in the overexpression lines (Figure 1A–C). It is interesting that, in comparison to WT rice plants, the content of *cis*-3-hexen-1-ol significantly rose in the R1, R6, and R8 lines, with increases of 3.98, 2.36, and 6.03 times, respectively (Figure 1A). Another GLV, *cis*-3-hexenal, was also significantly increased in these three *oeRCI* lines, with an increase of 2.41–4.37 times (Figure 1B). However, the content of *trans*-2-hexenal was only significantly increased in the R6 line and not in the R1 and R8 lines (Figure 1C). These results indicate that the overexpression of *OsRCI-1* may be involved in rice GLV production.

### 2.2. Effects of cis-3-hexen-1-ol and cis-3-hexenal on Feeding and Oviposition Preferences of BPH

**Figure 2:**
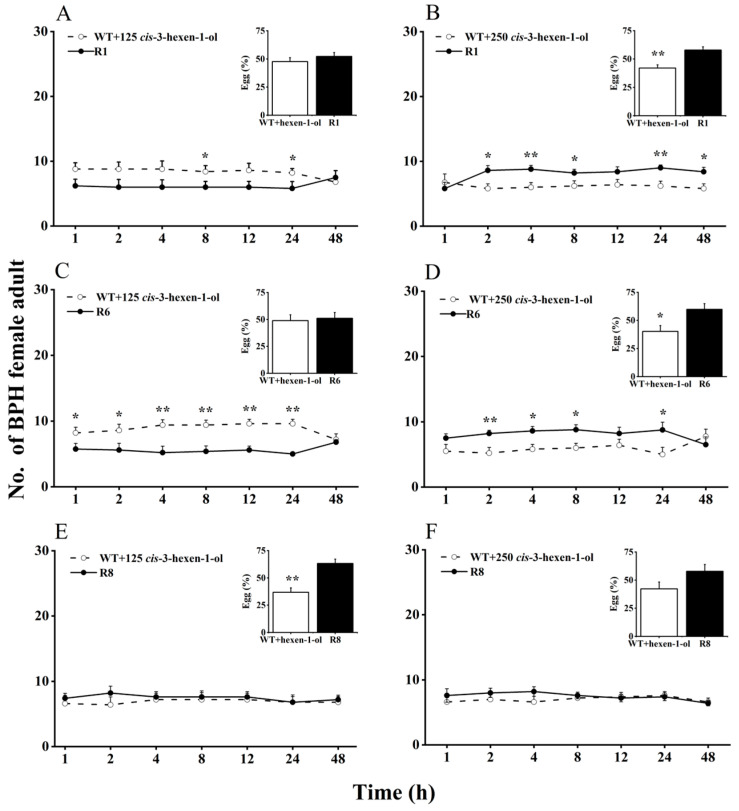
Effects of 125 (**A**,**C**,**E**) or 250 nmol (**B**,**D**,**F**) *cis*-3-hexen-1-ol on feeding and oviposition preference of BPH female adults (+SE, *n* = 5) in WT and *OsRCI-1* overexpression lines. Asterisks denote significant differences between pairs of groups within each time point (*, *p* < 0.05; **, *p* < 0.01; Student’s *t*-test).

**Figure 3:**
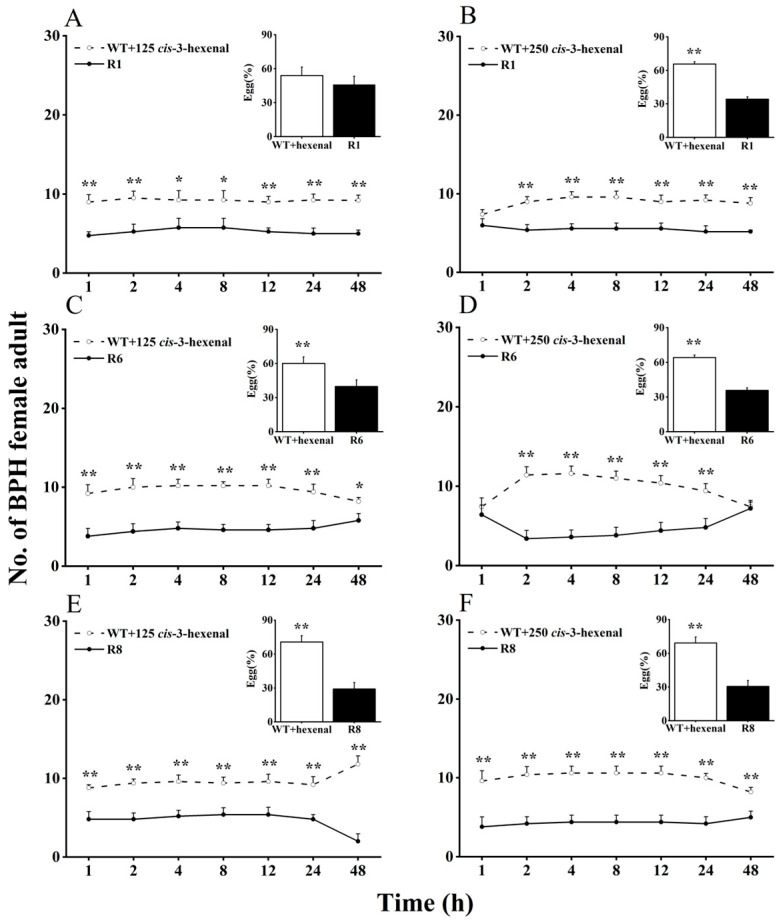
Effects of 125 (**A**,**C**,**E**) or 250 nmol (**B**,**D**,**F**) *cis*-3-hexenal on feeding and oviposition preference of BPH female adults (+SE, *n* = 5) in WT and *OsRCI-1* overexpression lines. Asterisks denote significant differences between pairs of members within time points (*, *p* < 0.05; **, *p* < 0.01; Student’s *t*-test).

Here, we evaluated BPH selection in WT rice plants supplemented with *cis*-3-hexen-1-ol or *cis*-3-hexenal, compared with *oeRCI* lines, to assess the activity of GLVs on BPH’s feeding and egg laying preference (Figure 2 and Figure 3). When 125 nmol of *cis*-3-hexen-1-ol was applied to the leaf sheaths of WT plants, BPH still showed a preference to feed and lay eggs on WT plants over *oeRCI* lines (R1 or R6) (Figure 2A,C). The preference of BPH was not evident in the comparison between *cis*-3-hexen-1-ol-treated WT plants and the R8 line (Figure 2E). In addition, when 250 nmol of *cis*-3-hexen-1-ol was applied to WT plants, a reversal in BPH’s feeding and oviposition preference was observed; BPH escaped feeding and oviposition on the R1 or R6 lines (Figure 2B,D). However, the application of either 125 or 250 nmol of another GLV, *cis*-3-hexenal, on the leaf sheaths of WT rice plants did not affect BPH, preferring *cis*-3-hexenal-treated WT plants (*p* < 0.05, Figure 3A–F) as well as untreated WT plants. These findings suggest that *cis*-3-hexen-1-ol but not *cis*-3-hexenal showed a repellent effect on the female adult BPH.

### 2.3. GLVs Modify Rice’s Attractiveness to Parasitoids after BPH Infestation

**Figure 4:**
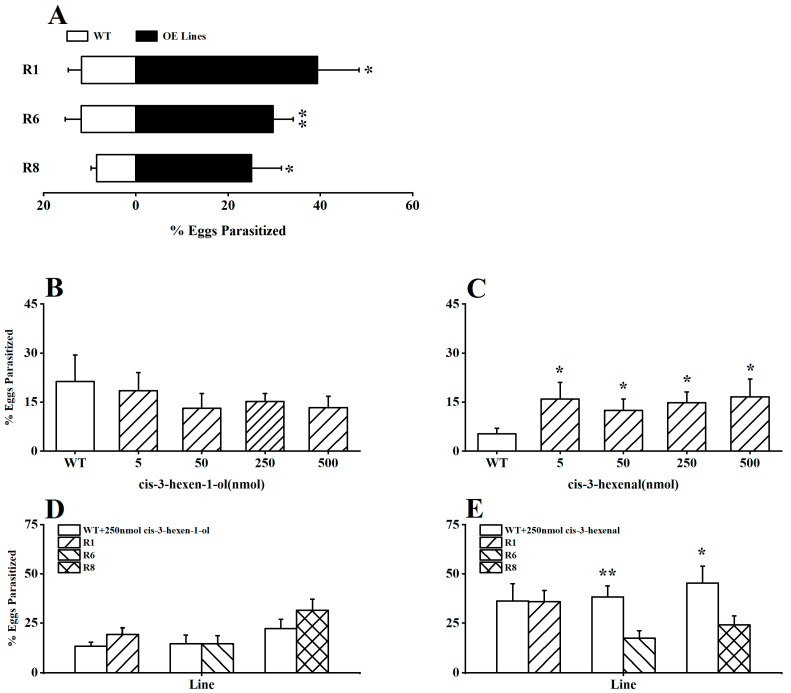
(**A**) The percentage (+SE, *n* = 7) of BPH eggs parasitized by *A. nilaparvatae* on R1, R6, R8, and WT plants. (**B**,**C**) The mean (+SE, *n* = 7) parasitism rates (%) of BPH eggs on WT plants treated with 5–500 nmol *cis*-3-hexen-1-ol or *cis*-3-hexenal in the field. (**D**,**E**) The mean (+SE, *n* = 7) parasitism rates (%) of BPH eggs in indoor experiments, with 250 nmol *cis*-3-hexen-1-ol or *cis*-3-hexenal applied to WT plants versus *oeRCI* lines. Asterisks indicate significant differences in R lines compared with WT plants (*, *p* < 0.05; **, *p* < 0.01; Student’s *t*-test).

To explore the potential impact of GLVs in rice on the parasitic preference of the parasitoid *A. nilaparvatae* (BPH egg parasitoid), we initially introduced the parasitoids into the *OsRCI-1* overexpression *oeRCI* lines and WT plants infested with BPH in a laboratory setting. It is interesting that the parasitism rates of BPH eggs on *OsRCI-1*-overexpressing plants were significantly higher than those on WT plants (*p* < 0.05), with the parasitism rates of BPH eggs on the R1, R6, and R8 lines being 3.35, 2.52, and 2.95 times higher than those on WT plants, respectively (Figure 4A). Furthermore, whether *cis*-3-hexen-1-ol or *cis*-3-hexen-al could increase the parasitism rates of BPH eggs on WT plants was assessed in the field. The application of *cis*-3-hexen-1-ol (5–500 nmol) on WT plants showed no significant difference in the parasitism rates of BPH eggs between treated and untreated WT plants (Figure 4B); however, the application of *cis*-3-hexenal at concentrations ranging from 5 to 500 nmol significantly elevated the parasitism rates of BPH eggs on WT plants in the field (*p* < 0.05; Figure 4C). The application of 250 nmol *cis*-3-hexen-1-ol to WT plants had no significant effect on the BPH egg parasitism rates in comparison to *oeRCI* lines in indoor experiments (*p* > 0.05; Figure 4D); unexpectedly, applying 250 nmol *cis*-3-hexenal to WT plants could deactivate or reverse *A. nilaparvatae*’s preference, parasitizing BPH eggs on *oeRCI* lines (Figure 4E), and the parasitism rates of BPH eggs on *cis*-3-hexenal-treated WT plants were apparently higher than those on the R6 and R8 rice plants (*p* < 0.05; Figure 4E).

## 3. Discussion

The expression of the *OsRCI-1* gene in rice can be rapidly and consistently induced by *Chilo suppressalis* and BPH infestation. *C. suppressalis* feeding on *OsRCI-1*-silenced lines increased in body weight by 1.58–2.15 times compared to feeding on WT plants [20]. The overexpression of *OsRCI-1* not only increased the levels of BPH-induced JA and JA-Ile but also decreased the feeding and oviposition preferences of BPH female adults, ultimately reducing BPH’s performance [18]. In this study, we found that the overexpression of *OsRCI-1* increased the levels of GLVs, including *cis*-3-hexen-1-ol and *cis*-3-hexenal. The increased GLVs, especially *cis*-3-hexenal and *cis*-3-hexen-1-ol (but not *cis*-3-hexenal alone), significantly regulated the BPH’s feeding and oviposition performance. Additionally, we observed that the wasp *A. nilaparvatae* preferred to parasitize BPH eggs on *OsRCI-1* overexpression rice plants. This preference of the wasp could be partly attributed to the increased levels of GLVs produced by the *OsRCI-1* overexpression plants. In conclusion, the rice lipoxygenase *OsRCI-1* is involved in GLV production of the HPL branch pathway, as well as the JA generation of the AOS branch pathway, in the rice jsamonate pathway, and the GLVs play an important role in protecting rice from invasive pests.

The different GLVs showed disparate biological functions in the interactions between plants, herbivores, and the enemies of herbivores. In the *OsRCI-1* overexpression lines, the levels of *cis*-3-hexen-1-ol and *cis*-3-hexenal but not *trans*-2-hexenal significantly increased, but it was found that *cis*-3-hexen-1-ol and *cis*-3-hexenal had different effects on the behavior of BPH and *A. nilaparvatae* (Figure 2, Figure 3 and Figure 4). The supplementation of *cis*-3-hexen-1-ol in WT rice plants could apparently alter the feeding and oviposition preferences of BPH adults (Figure 2), working as a repellent, and its repellent activity appeared to be dependent on its concentration (Figure 2). However, when adding *cis*-3-hexen-1-ol to WT rice plants in the field, no difference was found in the parasitism rates of BPH eggs on control rice and *cis*-3-hexen-1-ol-treated WT plants (Figure 4). Other studies showed that *cis*-3-hexen-1-ol is involved in regulating direct and indirect defense pathways in tea plants [21] and can activate defense responses against insect pests in plants like maize [22,23,24]. In contrast, *cis*-3-hexenal emitted from *OsRCI-1*-overexpressed rice lines did not change the feeding and oviposition preference of BPH adults (Figure 3), but significantly increased the parasitism rate of BPH eggs (Figure 4). Therefore, the rice lipoxygenase gene *OsRCI-1* could repel BPH by releasing *cis*-3-hexen-1-ol, functioning as a “push” strategy, and attract *A. nilaparvatae* by releasing *cis*-3-hexenal, therefore increasing the parasitism rate of BPH eggs, functioning as a “pull” strategy. Similar phenomena have been reported; Lamy et al. [25] found that the volatile dimethyl disulfide exhibited a “push” strategy by reducing the eggs laid on broccoli by the cabbage root fly (*Delia radicum* Linnaeus; Diptera: Anthomyiidae). Meanwhile, *cis*-3-hexenyl acetate exhibited a “pull” strategy by attracting the natural enemy of *D. radicum*.

The lipoxygenase pathway genes can regulate the emission of GLVs; therefore, they play an important role in the direct and indirect defense responses of rice. For example, compared to wild-type (WT) plants, mutants of antisense-suppressed phospholipase D (PLD) genes (*OsPLDa4* and *a5*) exhibited reduced levels of trypsin inhibitors and GLVs, specifically *cis*-3-hexenal and *cis*-3-hexen-1-ol [5]. This resulted in the increased growth and reproduction of BPH and *C. suppressalis*, along with decreased attraction to the natural enemy of *C. suppressalis*. Applying these two GLVs externally restored the resistance of the mutants to BPH and *C. suppressalis* [5]. The loss-of-function or antisense inhibition mutants of the rice hydrogen peroxide lyase gene (*OsHPL3*) exhibited reduced levels of *cis*-3-hexen-1-ol and *cis*-3-hexenal, resulting in decreased resistance to BPH but increased resistance against *WBPH*, and also increased attraction to the parasitoid *A. nilaparvatae* [8,26]. In this study, we found that the overexpression of the rice lipoxygenase gene *OsRCI-1* increased the levels of *cis*-3-hexen-1-ol and *cis*-3-hexenal (Figure 1), therefore reducing the feeding and oviposition preferences of BPH but enhancing the attraction of *A. nilaparvatae* (Figure 4E). These findings suggest that elevated levels of green leaf volatiles (*cis*-3-hexen-1-ol and *cis*-3-hexenal) contribute to enhancing the direct and indirect resistance of rice against BPH.

In summary, our study provides compelling evidence for the involvement of *OsRCI-1* in regulating the release of GLVs including *cis*-3-hexenal and *cis*-3-hexen-1-ol in rice; *OsRCI-1* is another LOX gene that contributes to both JA and GLV synthesis in rice as *OsLOX1* [16]. *OsRCI-1* overexpression in rice lines could significantly reduce BPH’s feeding and oviposition preferences and increase the attractiveness to egg parasitoid *A. nilaparvatae* [18]. Another new insight is the increase in *cis*-3-hexenal in rice plants, which functions as a repellent to BPH, and *cis*-3-hexen-1-ol in rice functions as an attractant towards the herbivore’s natural enemy. These results highlight the role of GLVs mediated by *OsRCI-1* in regulating both the direct and indirect defenses of rice against BPH. The rice lipoxygenase gene *OsRCI-1* and its overexpression in rice have potential applications in pest-resistant rice breeding.

## 4. Materials and Methods

### 4.1. Rice Materials

The rice genotype Xiushui 11 (WT) and three *OsRCI-1*-overexpressing transgenic *oeRCI* lines (R1, R6, and R8) [18] were used in this study. The rice seeds were first germinated in a growth chamber and then transplanted into 25-liter plastic pots filled with rice culture solution. Both seed germination and plant growth were monitored under controlled conditions (27 ± 2 °C, relative humidity 70–80%, and light–dark = 16:8). Rice seedlings at 35–40 days old were used for experiments.

### 4.2. Insect Materials

BPH populations were collected from rice fields in Lin’an, Zhejiang, China, and maintained on TN1 rice seedlings for at least ten generations in a controlled climate room at 27 °C ± 2 °C, 70–80% relative humidity, and a 12 h light phase, as reported by Liao et al. [18]. TN1 rice plants with BPH eggs were then placed in a rice field at Zhejiang A & F University to attract the parasitoid *A. nilaparvatae.* In the growth chamber, BPH female adults lay eggs on rice seedlings and then we offered these seedlings to the parasitoid.

### 4.3. Reagents and Instruments

The reagents used in this study were *cis*-3-hexen-1-ol, *cis*-3-hexenal, *trans*-2-hexenal, and lanolin, which were purchased from Shanghai Aladdin Biochemical Technology Co., Ltd., Shanghai, China. The instruments used were the RXZ-380B intelligent growth chamber (Ningbo Jiangnan Instrument Factory, Ningbo, Zhejiang, China) and the Motic binocular dissecting microscope (Meike Audi Industrial Group Co., Ltd., Xiamen, Fujian, China).

### 4.4. GLV Analysis

GLVs (*cis*-3-hexenal, *cis*-3-hexen-1-ol, and *trans*-2-hexenal) were analyzed with a portable gas analyzer (zNose 4200, Electronic Sensor Technology, Newbury Park, CA, USA) using the method described by Zhou et al. [6]. Rice leaves (0.02 g) from the three overexpression lines R1, R6, and R8 and their wild-type XS11 were cut into small pieces (about 2 mm long) and immediately placed in a 50 mL glass chamber (Jiangyin Elite Experimental Instrument Co., Ltd., Jiangyin, Jiangsu, China), respectively; after 5 min, GLVs were sampled for 40 s and analyzed by the zNose 4200. At least eight replicates were carried out.

### 4.5. BPH Bioassays

To estimate the impact of GLVs, specifically *cis*-3-hexen-1-ol or *cis*-3-hexenal, on the colonization and oviposition preference of BPH between the WT and oeRCI rice lines, pots with two plants were used. One plant was an *oeRCI* line treated with 10 μL lanolin and the other was a WT rice plant treated with 10 μL lanolin containing 125 or 250 nmol GLVs (*cis*-3-hexen-1-ol or *cis*-3-hexenal). The pots were confined within a glass cylinder (diameter, 4 cm; height, 8 cm; with 24 small holes (diameter 0.8 mm) evenly distributed on the cylinder wall) sealed with a sponge, into which 15 gravid BPH females were introduced. The number of BPHs on each plant was recorded at 1, 2, 4, 8, 12, 24, and 48 h. After 48 h, the number of BPH eggs on each plant was counted under a dissecting microscope. The experiment was repeated at least five times for each pair of plants.

### 4.6. Field Experiment

To evaluate the role of GLVs (*cis*-3-hexen-1-ol or *cis*-3-hexenal) in rice–parasitoid interactions in the field, WT plants (one plant per pot) were individually treated with 0, 5, 50, 250, or 500 nmol *cis*-3-hexen-1-ol or *cis*-3-hexenal in 10 μL lanolin and were infested with 8 BPH females for 24 h; then, the BPHs were removed as described by Liao et al. [19]. Five potted plants (each of 0, 5, 50, 250, or 500 nmol *cis*-3-hexen-1-ol- or *cis*-3-hexenal-treated WT plants) were placed and arranged within a 40 cm circular radius as located in a rice field. The location was 1 m from the edge of the rice field, and the distance between each location was kept at 2 m. Two days later, the plants were transferred to a controlled climate room at 27 ± 2 °C with a 14 h light phase and 70% relative humidity for five days. Then, the total number of BPH eggs and eggs parasitized by the wasps (these eggs became red) were counted. Each treatment was repeated at least seven times.

### 4.7. Wasp Bioassays in Controlled Climate Chamber

The WT and *oeRCI* rice lines were infested with 12 BPH females and oviposited for 12 h, respectively, and then the BPHs were removed. Pots with two plants (one *oeRCI* line with BPH eggs vs. a WT plant with BPH eggs) were kept 1 cm apart and were confined within glass cylinders (diameter 4 cm, height 8 cm, with 24 small holes), into which six newly emerged *A. nilaparvatae* wasps (3♀ and 3♂) were introduced, as described by Liao et al. [19]. After 24 h, the parasitoid wasps were removed, and the rice plants were kept in a controlled climate room at 27 ± 2 °C with a 14 h light phase and 70% relative humidity for five days. Then, the total number of BPH eggs and eggs parasitized by the wasps were counted. Each treatment was repeated at least seven times.

## Data Availability

Data are contained within the article.
